# Emergencies in inflammatory rheumatic diseases

**DOI:** 10.1007/s00296-024-05660-y

**Published:** 2024-07-09

**Authors:** Dana Auyezkhankyzy, Aigulsum Izekenova, Burhan Fatih Kocyigit

**Affiliations:** 1 Department of Emergency Medicine and Nursing, South Kazakhstan Medical Academy, Shymkent, Kazakhstan; 2 Department of Epidemiology with the Course of HIV Infection, Asfendiyarov Kazakh National Medical University, Almaty, Kazakhstan; 3 Department of Physical Medicine and Rehabilitation, University of Health Sciences, Adana City Research and Training Hospital, Adana, Türkiye

**Keywords:** Rheumatic diseases, Emergencies, Crisis intervention, Critical care, Emergency treatment, Rheumatism

## Abstract

Inflammatory rheumatic diseases (IRDs), encompassing a broad spectrum of chronic disorders, typically necessitate prolonged therapeutic intervention. Nevertheless, these diseases can sometimes manifest as severe emergencies requiring prompt and extensive medical intervention. Urgent intervention is essential for effectively recognizing and managing these situations, as they have the potential to be life-threatening and can result in severe morbidity and mortality. Emergencies in IRDs can occur with different frequencies and manifestations, including nervous system issues, severe infections, thrombosis-emboli, renal crises, gastrointestinal issues, and cardiovascular events. The fact that these events can occur across different IRDs underscores the necessity for heightened awareness and readiness among healthcare professionals. The pathophysiologic mechanisms that cause rheumatic emergencies are complex and involve multiple factors. These emergencies frequently arise due to the interplay between the inflammatory characteristics of rheumatic diseases and different systemic triggers. Early detection and treatment can have a substantial impact on an individual’s prognosis in cases of severe and life-threatening disorders that require prompt recognition. Rapid decision-making and urgent care are required to effectively address rheumatic emergencies, as well as the implementation of a diagnostic flowchart. This article provides an overview of the emergencies linked to IRDs, classifying and assessing them individually. This article aims to enhance healthcare professionals’ knowledge and awareness of critical situations by examining current recommendations and pathophysiological information. Implementing standardized diagnostic and treatment methods, providing patient education, and conducting continuing research into the underlying mechanisms are essential for enhancing the management of these critical situations and improving patient outcomes.

**Supplementary Information:**

The online version contains supplementary material available at 10.1007/s00296-024-05660-y.

## Introduction

Inflammatory rheumatic diseases (IRDs), encompassing a broad spectrum of disorders, typically exhibit a chronic nature and necessitate prolonged therapeutic interventions. Nevertheless, these disorders can sometimes manifest as acute emergencies necessitating immediate and intensive medical intervention. It is crucial to acknowledge and efficiently handle these emergencies in rheumatology, as they have the potential to be life-threatening and can result in severe morbidity and death if not promptly addressed [1, 2].

Emergencies in IRDs differ in frequency and presentation. These situations involve, but are not limited to, severe infections [3], pulmonary emboli [4], renal crises [5], and cardiovascular issues [6]. Such events are relatively common across IRDs, underlining the need for enhanced awareness and readiness among healthcare professionals. Early identification and management can significantly influence the individual’s prognosis in cases of severe and life-threatening conditions that require swift recognition. Prompt decision-making and urgent care are necessary to effectively handle rheumatic emergencies, along with a diagnostic flowchart [7].

The pathophysiological processes that cause rheumatic emergencies are intricate and have multiple aspects. These consequences frequently arise from the interaction between the inflammatory nature of rheumatic disease and multiple systemic factors. Active inflammation can cause a hypercoagulable condition, which increases the risk of thromboembolic events. Additionally, immunosuppressive drugs can make patients more susceptible to severe infections. A comprehensive understanding of these mechanisms is crucial for developing precise therapeutic interventions and successfully implementing efficient management approaches [8, 9].

This article presents a comprehensive overview of the several emergencies observed in the context of IRDs. IRDs are categorized and discussed separately, describing the potential emergencies that may arise from these diseases. By examining current recommendations and pathophysiological details, this article seeks to enhance physicians’ understanding of efficiently identifying and addressing crucial circumstances.

## Search strategy

Relevant publications were retrieved from Web of Science, Scopus, Medline/PubMed, Scopus, and DOAJ by conducting searches using the terms “emergencies” or “critical care” and “rheumatic diseases” or “rheumatology” or “rheumatoid arthritis” or “systemic lupus erythematosus” or “Sjogren’s syndrome” or “spondyloarthritis” or “systemic sclerosis” or “Behcet disease” or “familial Mediterranean fever” or “myositis” or “systemic vasculitis”. Only English items that were published before June 2024 were considered. No specific time window was determined. The examination encompassed all sorts of articles. We also made an effort to carefully review the references included in the articles that were discovered using our search strategy and choose the ones that we thought were relevant. The search was carried out according to the parameters laid forth by Gasparyan et al. [10].

## Rheumatoid arthritis

Atlantoaxial subluxation is a severe consequence of rheumatoid arthritis (RA). The condition is characterized by the incomplete displacement or misalignment of the first (atlas) and second (axis) cervical vertebrae. The disease’s extended duration and structural alterations make surfaces of joints and ligaments less stable, hence heightening the susceptibility to damage. Forward dislocation is more frequent, but vertical dislocation is more severe. Subluxation can lead to respiratory arrest as a result of compression of the spinal cord. The patient’s medical history and results from the physical examination reveal the presence of recent occipital discomfort, feeling paresthesias, sensory disruptions, extremity weakness, dizziness, an experience of electric shock while flexing the cervical spine, and issues with the urinary system [11, 12]. Cervical affections were associated with involvement in hand and foot radiographs and RA diagnosis before the age of 45 years [13]. Studies have shown a quite diverse prevalence of cervical involvement among RA patients, varying from 25 to 88% [14]. Radiologic evaluation is crucial for evaluating the alignment and potential involvement of the spinal cord. Regularly monitoring those patients is essential, as prompt identification can avert serious consequences [15]. Conservative treatment involves implementing preventive measures, minimizing excessive neck movement, and utilizing cervical collars. If the condition is severe, surgical intervention may be required to stabilize the spine and avoid any potential neurological harm [16].

Rheumatoid arthritis (RA) frequently presents with cardiac emergencies, including myocardial infarction, pericarditis, myocarditis, and arrhythmias [17]. RA patients have a two times greater risk of atherosclerotic cardiovascular disorders than the general population. Early RA patients have a higher risk of ischemic cardiac events, maybe even in the asymptomatic stage, and they also have an almost twofold higher risk of congestive heart failure [18]. Early detection and management of these conditions can prevent negative consequences. Pleuritis is a frequently occurring pulmonary consequence of RA. Patients exhibit symptoms such as difficulty breathing, chest pain, fluid accumulation, or the detection of a rubbing noise during examination. Therapeutic thoracentesis treats severe effusions that cause symptoms to avoid scarring or fibrosis [11]. Pneumonia is a frequently occurring disease in RA due to the immunosuppressive effects of anti-rheumatic drugs and the disease itself. While individuals in this specific subset will still be exposed to the same disease-causing agents as the general population, it is the opportunistic organisms that are primarily responsible for sepsis and the high rates of morbidity and death in these patients [11, 19, 20].

Acute nephritis, rhabdomyolysis, renal vein thrombosis, and fulminant renal failure can occur as a concomitant of the disease process and can be drug-induced [21]. Gastrointestinal hemorrhage is a prevalent observation in RA. It can be caused by conditions such as gastrointestinal ischemia, ulcerations, and the side effects of pharmacological therapy [22].

Felty syndrome is distinguished by neutropenia, splenomegaly, and recurrent bacterial infections. It often manifests in patients with persistent, seropositive conditions characterized by severe joint damage. The predicted prevalence of this condition in individuals with RA is 1–3% [23]. In most cases, it becomes apparent as a clinical picture after the disease has progressed considerably. It is crucial to promptly address infection and combat neutropenia during emergencies [24].

## Systemic lupus erythematosus

Lupus nephritis is an inflammatory condition of the kidneys, which can result in either acute or chronic renal failure [25]. The rapid deterioration of renal function accompanied by an active presence of urine sediment and an elevated protein level in the urine is a cause for concern, as it may indicate rapidly progressive glomerulonephritis. Hereditary susceptibility, female gender, smoking, chemical and UV exposure, and infections are among the risk factors for lupus nephritis [26]. This condition necessitates immediate assessment and treatment. Investigating other factors that can cause the kidneys to decline function, such as acute tubular necrosis, is essential. This is particularly relevant if an individual has recently used nonsteroidal anti-inflammatory drugs [27, 28].

Among various clinical features, systemic lupus erythematosus (SLE) affects the nervous system, producing a variety of complaints in both the peripheral and central nervous systems. A serious complication of SLE, neuropsychiatric SLE is defined by neurological and psychiatric signs [29]. Neuropsychiatric SLE can present as either localized or diffuse, with a wide range of clinical symptoms, including cognitive impairment, severe disorientation, seizures, and psychotic signs. Furthermore, headaches, mood disturbances, and intellectual disabilities are the predominant neuropsychiatric symptoms observed in SLE. The most prevalent neuropsychiatric manifestations linked to SLE include stroke, neuropathies, rapid confusional states, and epileptic seizures [30, 31].

Diffuse alveolar hemorrhage is a severe pulmonary issue that can occur in SLE. The condition progresses over hours to many days and is the syndrome linked to SLE with a high death rate. While no particular signs are mentioned, diffuse alveolar hemorrhage is typically characterized by several aspects, the most notable being a decrease in hemoglobin levels. Other frequently reported symptoms include breathing difficulties, bloody sputum, diffuse infiltrates seen by chest scans, thrombocytopenia, and hypocomplementemia [32].

Catastrophic antiphospholipid syndrome is a highly severe manifestation of this disease, characterized by extensive blood clot formation that can fail numerous organs. The clinical presentation involves the abrupt development of organ dysfunction, such as kidney failure, breathing difficulties, and neurological signs [33]. This condition has been linked with a mortality rate of around 50%. The primary consequences of the disorder are infections, failure of multiple organs, and cranial and heart thromboembolism [34].

Cardiac tamponade in SLE is related to pericardial fluid accumulation and decreased heart function. Although pericarditis is relatively common in SLE patients, cardiac tamponade is much rarer, with an incidence of < 2.5% [35]. Relevant findings include chest pain, difficulty breathing, low blood pressure, tachycardia, and swollen neck veins. Urgent echocardiography and, if necessary, pericardiocentesis to remove fluid are recommended [36].

Macrophage activation syndrome is a serious and sometimes fatal condition mostly linked to systemic juvenile idiopathic arthritis. It may also manifest in various rheumatic conditions, including SLE, Kawasaki disease, Still’s disease, and juvenile dermatomyositis. This condition is distinguished by an overactive and rapid growth of macrophages and T lymphocytes, resulting in excessive inflammation. Macrophage activation syndrome is classified as a type of secondary hemophagocytic lymphohistiocytosis. The following important features are involved: hemophagocytosis, immune cell activation, and cytokine storm. The clinical manifestation may be sudden and intense, characterized by symptoms such as high body temperature, liver and spleen enlargement, cytopenia, excessive ferritin levels, coagulopathy, increased levels of liver enzymes, and neurological signs. The treatment needs immediate and proactive intervention to regulate the hyperinflammatory condition. Treatment involves the use of corticosteroids and immunosuppressive drugs. Providing supportive care and addressing the condition’s root cause are crucial [37, 38].

## Sjögren’s syndrome

Neurological issues are a recognized occurrence in Sjögren’s syndrome (SS), with approximately 2–5% of SS cases involving the central nervous system and an even larger percentage affecting the peripheral nervous system [39]. The involvement of the central nervous system encompasses several conditions, such as demyelinating lesions mimicking multiple sclerosis, transverse myelitis, lymphocytic meningitis, vasculitis, neuromyelitis optica, and acute encephalopathy [40, 41]. Neurologic complications in SS can be predicted by decreased complement concentrations, xerophthalmia, antinuclear antibody positivity, cardiac commitment, and a positive salivary gland biopsy finding [42]. Patients may be admitted to the emergency department with sudden onset of neurologic impairments, mental status changes, seizures, and focal neurologic abnormalities.

Renal tubular acidosis is a particular issue that occurs in individuals with renal involvement due to SS. It is characterized by metabolic acidosis, urinary stones, bone disease, weakness in the muscles resulting from hypokalemia, and, in severe cases, respiratory failure and cardiac arrest. Timely detection is the crucial initial measure to avert negative outcomes such as bone fractures, potentially fatal muscular paralysis, and chronic renal disease [43].

Systemic vasculitis, although uncommon, is a highly severe form of SS that occurs regardless of the glands and contributes to the increasing morbidity and mortality associated with the disease. Ischemia of the tissues and organ damage occur in conjunction with vasculitis. Patients can arrive at the emergency room with organ dysfunction or failure, evident purpura, neurologic impairments, and wounds on the skin [44].

The frequency of lymphoma and interstitial lung disease is increasing in SS. Lymphoma formation has been linked to inherited characteristics, involvement of the salivary glands, lymphopenia, cryoglobulinemia, low levels of complement, and substantial lymphocyte involvement in salivary gland histology [42]. Interstitial lung disease is an entity that can be associated with severe morbidity and mortality, with prevalences as high as approximately 20% in SS patients [45]. SS patients can present to the emergency department concerning these conditions: fever, fatigue, dyspnea, cough, and hypoxia [46, 47].

## Spondyloarthritis

Patients with advanced spondyloarthritis (SpA) have a high risk of spinal fractures due to persistent inflammation, syndemophytes, and spine ankylosis. These fractures can occur following a minor trauma [48]. The spine becomes fragile as a result of secondary osteoporosis and reduced mobility associated with SpA. As the condition progresses, the fracture risk gradually rises [49]. Various research has investigated spinal fractures in SpA, revealing that the prevalence of fractures using radiographic imaging ranges from 1.4 to 58.0%. Nevertheless, the criteria for enrolling patients differed among the Research [50]. Patients present with severe back pain, usually in the form of sudden, intense pain at the fracture site. Neurological deficits, depending on the location, numbness, weakness, or paralysis may be present due to spinal cord or nerve root compression [51]. Immediate immobilization and radiological assessment are necessary. Treatment may involve surgical stabilization and pain control, particularly in cases where there are neurological impairments [52].

Cauda equina syndrome is an infrequent neurological condition that can arise as a result of long-term SpA [53]. The nerve roots at the distal region of the spinal cord tend to be affected, leading to sensory and/or motor impairments in the pelvic region and lower extremities, which can include bladder and bowel function issues. This primarily results from bone and soft tissue alterations in the spine that compress the cauda equina nerve roots [54]. This situation is a critical medical emergency that necessitates urgent surgical decompression in order to avoid long-lasting neurological harm. Postoperative care involves the implementation of rehabilitation and the careful observation of potential issues [55].

## Systemic sclerosis

Scleroderma renal crisis is a severe complication of systemic sclerosis (SSc). Data on the frequency and affecting facets of scleroderma renal crisis have always had constraints. This results from the fact that it is an uncommon event in a rare disease with varied presentations and continuous discussion on an appropriate diagnosis. Hypertensive encephalopathy, seizures, and acute heart failure are commonly linked to rapidly progressing hypertension. Renal function decline is frequently observed and often necessitates dialysis. Schistocytes are seen in a peripheral smear due to microangiopathic hemolytic anemia, characterized by the destruction of red blood cells and thrombocytopenia. Immediate administration of angiotensin-converting enzyme inhibitors (ACE inhibitors) is crucial for emergency treatment. ACE inhibitors can effectively manage blood pressure and enhance outcomes, even in cases of acute renal injury. Providing supportive care and necessary medical treatment, such as dialysis, is critical [56–58].

Pulmonary arterial hypertension is a prevalent cause of morbidity and mortality in SSc. Potential risk factors for pulmonary arterial hypertension include the presence of positive anticentromere antibodies, advanced age, prolonged disease, and the coexistence of interstitial lung disease. However, it should be noted that there can be discrepancies between research. Acute severe dyspnea can manifest as sudden shortness of breath, frequently caused by physical activity. Presentation to the emergency department can arise from syncope, inadequate cardiac output, and episodes of chest pain and fainting [59–61].

Interstitial lung disease is frequently observed in SSc, and sudden exacerbations can result in respiratory failure. Up to 80% of patients with systemic sclerosis have been diagnosed with interstitial lung disease based on high-resolution computed tomography of the lung, and up to 90% have been confirmed by biopsy assessments. Nevertheless, a lower rate of individuals, ranging from 30 to 40%, experience a notable clinical presentation. The clinical presentation consists of a rapid deterioration in breathing difficulty, low oxygen levels, and diffuse alveolar damage. Exacerbations can be caused by infections, aspiration, or fibrosis growth, resulting in intense inflammation and injury to the alveolar-capillary membrane [62–64].

Emergencies in SSc necessitate a significant level of clinical focus and prompt action. To correctly manage these life-threatening issues, it is crucial to comprehend the fundamental pathophysiologic mechanisms and adhere to established treatment standards. Early identification and personalized therapeutic approaches are crucial for enhancing results and decreasing fatality rates in patients with SSc.

## Familial Mediterranean fever

Familial Mediterranean fever (FMF) is a critical condition when screening individuals with acute abdominal pain, particularly those from the Eastern Mediterranean region. Differentiating acute episodes from other causes of acute abdomen, such as acute appendicitis, cholecystitis, and intestinal obstruction, can be challenging. Radiologic findings during acute episodes are not specific. However, imaging can help rule out other possible causes of acute abdomen. It is essential to carry out a comprehensive patient assessment in emergency departments. Surgical intervention is typically unnecessary unless complications emerge [65, 66].

Acute chest pain can occur in FMF patients due to pleuritis and pericarditis. Symptoms may manifest as sharp chest pain, fever, and difficulty breathing. This clinical picture may refer healthcare professionals to urgent cardiac pathologies in the emergency department. Detailed anamnesis, physical examination, and laboratory investigations will provide a correct spectrum of approaches [67].

Acute inflammatory attacks that affect the abdomen, chest, and other structures, as well as potential long-term complications such as amyloidosis, are the primary concerns of FMF emergencies. FMF patients may occasionally exhibit chronic inflammation, characterized by consistently increased acute-phase reactant levels during periods without attacks. This condition is thought to be a predisposing factor for the long-term development of amyloidosis [68]. Multiple variables contribute to the development of FMF-associated amyloidosis. These factors consist of the male sex, the presence of arthritis, prolonged diagnostic procedures, the M694V homozygous genotype, and a familial background with confirmed amyloidosis [69].

Implementing prompt diagnosis, appropriate management, and other supportive therapies is essential to preventing severe repercussions and maintaining patients’ quality of life.

## Behcet disease

Neurologic involvement is an important contributor to disability and mortality in Behcet disease (BD). Even though neurologic involvement in BD typically manifests after a particular period following the onset of other systemic findings, it is uncommon for it to be the first sign of involvement [70]. Disruptions to the central and peripheral nervous systems are potential consequences of neurologic involvement. There are two main types of involvement in the central nervous system: parenchymal and non-parenchymal. The majority of cases are of the parenchymal type. Cerebral venous sinus thrombosis and artery involvement are instances of non-parenchymal involvement [71]. Patients exhibit symptoms such as headache, neurological impairments, seizures, dizziness, confusion, and alterations in behavior [72]. Treatment frequently necessitates a multidisciplinary approach that includes neurologists and rheumatologists, and if not treated promptly, can result in long-term neurological deficits and higher mortality rates.

Vascular commitment is a major clinical characteristic of BD, marked by repeated inflammatory thrombosis that affects the veins and, less commonly, the arteries. Superficial and deep vein thrombosis are the predominant forms of vascular participation. The superior vena cava, inferior vena cava, portal structures, suprahepatic veins, and right ventricle are implicated. Venous thrombosis frequently occurs in conjunction with aneurysms [73, 74]. Vascular involvement can result in life-threatening circumstances, including severe ischemia, arterial rupture, and massive pulmonary embolism. Disease management necessitates immunosuppressive therapy. It has been demonstrated to decrease the recurrence rate and extend the survival duration [75].

Gastrointestinal involvement is present in BD, and associated emergencies may occur. The predominant complaints are abdominal pain of varied severity, diarrhea with or without hemorrhage, and fever. Identifying characteristic ulceration during colonoscopy is crucial for making a diagnosis. The histopathologic analysis of surgical specimens reveals the presence of neutrophils infiltrating the tissues [76]. Patients with BD may present to emergency departments with clinical pictures of perforation, bleeding, ischemia, and infarction. Urgent radiological examination and medical treatment are planned. Surgical intervention can be performed in cases of perforation or severe bleeding [77].

## Inflammatory myositis

An emergent picture can result from fast-growing muscular paralysis, dysphagia (trouble in swallowing), and respiratory muscle involvement, leading to dyspnea in inflammatory myositis. Swallowing is an intricate neuromuscular activity that necessitates accurate motor synchronization. Inflammatory myositis frequently impacts the upper part of the esophagus and oropharynx, which are composed of striated skeletal muscle structures. Consequently, it is anticipated that myositis can lead to dysphagia by causing inflammation in the muscles responsible for swallowing. Dysphagia can result in aspiration pneumonia. Respiratory failure can emerge due to the impairment of the diaphragmatic and intercostal muscles. Furthermore, acute respiratory failure can arise as a result of the progression of interstitial lung disease. The frequency of interstitial lung disease varies substantially, ranging from 20 to 86%, based on the diagnostic method and the particular forms of inflammatory myopathy being investigated. In addition to disease-specific anti-rheumatic treatment, patients are provided with supportive care, which involves nasogastric nutrition to prevent aspiration and respiratory support (mechanical ventilation if required) [78–81].

Dysphagia is the most frequent gastrointestinal consequence. Manometric disorders may manifest. The findings are weak contractions of the esophageal body exhibiting low amplitude, ineffective peristalsis, and reduced pressure in the upper esophagus sphincter. Occasionally, individuals can exhibit ulcers, bleeding, perforation, and intestinal pneumatosis. In addition to medical treatment, nutritional support is offered through nasogastric or percutaneous endoscopic gastrostomy feeding. When required, a surgical opinion is obtained [82].

## Systemic vasculitis

Systemic vasculitis can lead to acute renal failure and necessitate dialysis. Renal issues are present in 25–75% of individuals with antineutrophil cytoplasmic antibody-associated systemic vasculitis, and the extent of kidney function at the time of diagnosis is a strong indicator of survival rates. Emergency management is essential for patients experiencing rapidly progressive glomerulonephritis, along with signs such as hematuria, proteinuria, hypertension, and renal failure. The treatment approach employs medical treatment, dialysis, and emergency plasma exchange according to the patient’s status [83, 84].

Diffuse alveolar hemorrhage is a critical, occasionally fatal clinical disorder characterized by the buildup of red blood cells in the alveolar area. It necessitates thorough intervention. Diffuse alveolar hemorrhage in vasculitis is the result of capillary inflammatory processes. The primary characteristics of this condition include difficulty in breathing, hemoptysis, abnormal infiltrates in the chest, and a sudden decrease in the quantity of hemoglobin value. A bronchoalveolar lavage showing a vibrant red liquid is a valuable diagnostic indicator that also rules out infection and other possible reasons for hemoptysis. A supportive treatment strategy is necessary. In serious situations, ventilatory support and extracorporeal membrane oxygenation can be utilized [85].

Systemic vasculitis can lead to the impact of the central nervous system. The involvement of the central nervous system presents clinical challenges due to non-specific clinical signs, the difficulty in obtaining pathological samples to confirm the diagnosis, the absence of effective non-invasive diagnostic procedures, and the relatively low frequency of their occurrence [86]. Patients have symptoms including intense headaches, focal neurological impairments, seizures, changes in mental state, and stroke-like clinic [87]. The patient is managed with confirmation of central nervous system involvement by imaging, specific medical treatment, and neurology consultation when necessary.

Gastrointestinal signs rarely characterize systemic vasculitis; however, they can swiftly escalate to life-threatening stages. The presence of gastrointestinal complications in large vessel vasculitis is infrequent and mainly results from the narrowing and blockage of large vessels, leading to symptoms of tissue ischemia. Small vessel vasculitis can result in various gastrointestinal signs, including mucosal involvement, granulation, necrotic ulcers, and possibly perforation. Gastrointestinal complications, including ischemia, severe bleeding, and perforation, can occur as a result of systemic vasculitis. Emergency imaging and a targeted medical strategy are utilized. If deemed required, surgical intervention is conducted [88, 89].

## Conclusion, perspectives, and recommendations

Emergencies in IRDs are a critical part of rheumatology that necessitates prompt diagnosis and treatment. If not handled immediately, these conditions may deteriorate rapidly and cause significant morbidity and mortality. Each IRD poses distinct obstacles with occasionally life-threatening consequences, emphasizing the importance of healthcare professionals maintaining high suspicion and being ready to act promptly. Emergencies are summarized in Table 1.

**Table 1:** Summarizing of potential emergencies in inflammatory rheumatic diseases

Rheumatic disease	Potential emergencies^*^
Rheumatoid arthritis	• Atlantoaxial subluxation • Cardiac disorders • Renal disorders, including fulminant renal failure • Felty syndrome • Gastrointestinal hemorrhage • Sepsis • Pneumonia such as pneumocystis pneumonia
Systemic lupus erythematosus	• Lupus nephritis and renal failure • Neuropsychiatric involvement • Diffuse alveolar hemorrhage • Catastrophic antiphospholipid syndrome • Cardiac tamponade • Macrophage activation syndrome
Sjögren’s syndrome	• Neurologic involvement • Renal tubular acidosis • Vasculitis-related problems • Lymphoma related problems • Interstitial lung disease-related emergencies
Spondyloarthritis	• Spinal fractures and neurologic deficits • Cauda equina syndrome
Systemic sclerosis	• Renal crisis • Pulmonary arterial hypertension-related signs • Interstitial lung disease-related emergencies
Familial Mediterranean fever	• Distinguishing acute abdominal pain and acute chest pain from other possible emergencies
Behcet disease	• Neurologic involvement • Inflammatory thrombosis and other vascular involvements • Gastrointestinal involvement (perforation, bleeding, ischemia, and infarction)
Inflammatory myositis	• Fast-growing muscular paralysis, dysphagia, and respiratory muscle involvement • Gastrointestinal involvement (ulcers, bleeding, perforation, and intestinal pneumatosis)
Systemic vasculitis	• Renal failure and necessitate dialysis • Diffuse alveolar hemorrhage • Neurologic involvement • Gastrointestinal complications, including ischemia, severe bleeding, and perforation

^*^ Potential emergencies include disease-related situations and side-adverse effects of medical treatment approaches

Standardized diagnostic and treatment approaches can assist in the early detection and management of acute crises. Furthermore, advancements in imaging techniques and biomarker discoveries may help to predict and diagnose issues earlier.

Exploring the underlying pathophysiologic processes that produce emergencies in IRDs is critical for developing targeted therapeutics. Furthermore, patient education and awareness are vital components in treating IRDs. Enabling patients to recognize early indicators of issues and seek prompt medical assistance can substantially impact their prognosis and quality of life.

In conclusion, although IRDs are primarily chronic, the potential for acute, life-threatening emergencies necessitates meticulous monitoring and immediate, decisive action. The medical community can enhance the management of these critical situations and ultimately improve the outcomes for individuals with IRDs by promoting a multidisciplinary approach, furthering research, and emphasizing patient education.

### Electronic supplementary material

Below is the link to the electronic supplementary material.

Supplementary Material 1

Supplementary Material 2

Supplementary Material 3

Supplementary Material 4

Supplementary Material 5

Supplementary Material 6

## Declarations

### Disclaimer

No part of this article is copied or published elsewhere in whole or in part in any languages.
